# Neuroinflammation, Neuroautoimmunity, and the Co-Morbidities of Complex Regional Pain Syndrome

**DOI:** 10.1007/s11481-012-9392-x

**Published:** 2012-08-25

**Authors:** Mark S. Cooper, Vincent P. Clark

**Affiliations:** 1 Department of Biology, University of Washington, Seattle, WA 98195-1800 USA; 2 Departments of Psychology and Neurosciences, University of New Mexico, Albuquerque, NM 87131-0001 USA; 3 Mind Research Network and Lovelace Biomedical Research Institute, Albuquerque, NM 87106 USA

**Keywords:** Neuropathic pain, Psychogenic, Physiologic, Hysteria, Somatoform, Conversion disorder, Microglia, GABAergic, Glycinergic, Neuroimaging, Reflex sympathetic dystrophy, Charcot, Autoimmune, Neuroautoantibodies, Neuroautoimmunity

## Abstract

Complex Regional Pain Syndrome (CRPS) is associated with non-dermatomal patterns of pain, unusual movement disorders, and somatovisceral dysfunctions. These symptoms are viewed by some neurologists and psychiatrists as being psychogenic in origin. Recent evidence, however, suggests that an autoimmune attack on self-antigens found in the peripheral and central nervous system may underlie a number of CRPS symptoms. From both animal and human studies, evidence is accumulating that neuroinflammation can spread, either anterograde or retrograde, via axonal projections in the CNS, thereby establishing neuroinflammatory tracks and secondary neuroinflammatory foci within the neuraxis. These findings suggest that neuroinflammatory lesions, as well as their associated functional consequences, should be evaluated during the differential diagnosis of non-dermatomal pain presentations, atypical movement disorders, as well as other “medically unexplained symptoms”, which are often attributed to psychogenic illness.

Hysteria has its laws, its determination, precisely like a nervous system ailment with a material lesion. Its anatomical lesion still eludes our means of investigation…” Dr. Jean-Martin Charcot, 1890.

## Introduction

Complex Regional Pain Syndrome (CRPS), formerly referred to as Reflex Sympathetic Dystrophy (RSD), is one of the diseases classically defined as *hysteria minor* by the early neurologist, Dr. Jean-Martin Charcot (1892). To this day, the sensory disorders and movement disorders of CRPS are sometimes diagnosed as somatization disorders, or conversion disorders, respectively (Ochoa and Verdugo 1995; Verdugo and Ochoa 2000; Hawley and Weiner 2011). Such somatoform disorders are defined as a chronic condition where physical symptoms are observed, but no physical cause can be found (Stone et al. 2011). In the absence of medical explanations for the symptoms, a psychological etiology is presumed (Stone et al. 2009, Stone et al. 2010).

In contrast to these views, substantial evidence has been obtained that CRPS is a neuroinflammatory disorder, with a probable autoimmune component in many individuals (Blaes et al. 2007, Goebel et al. 2011; Kohr et al. 2011; Goebel 2011). In a study of adult CRPS patients, 90% of the cohort had autoantibodies to either the beta(2)-adrenergic receptor (β2AR) or the muscarinic acetylcholine receptor (M2R) (Kohr et al. 2011). 55% of the patients had autoantibodies to both neurotransmitter receptors. Integrating these new research findings into neurological and psychiatric practice will require a comparison between historical views on hysteria, contemporary views on psychogenic illness, as well as emerging information about neuroautoimmunity and neuroinflammation.

In the 1880s, Charcot first hypothesized that hysteria was generated by non-structural lesions in the nervous system (Harris 2005). He postulated that these lesions were likely to be biochemical or physiological in character. In describing a case study, during a lecture on hysteria, Charcot (1885) stated: “We have here unquestionably one of those lesions which escape our present means of anatomical investigation, and which, *for want of a better term, we designate dynamic or functional lesions*.”

Charcot’s concepts of dynamic and functional lesions are especially useful to utilize when analyzing the local and non-local effects of neuroinflammation within the nervous system. Neuroinflammation is now implicated in many neurological and neuropsychiatric disorders that were once classified as hysteria, e.g. dystonia (Sigel et al. 2007; Lee et al. 2009; Simonyan et al. 2010), CRPS (Del Valle et al. 2009), temporal lobe epilepsy (Huberfeld et al. 2007). In this paper, we discuss how *neuroinflammatory lesions*, together with their functional consequences, are likely to be a subset of the functional and dynamic lesions that were originally postulated by Charcot to explain the etiology of hysteria (1885). In addition, we discuss the need to improve neuroimaging of neuroinflammatory lesions, to help facilitate the diagnosis and mechanistic understanding of neuroinflammation-mediated neurological and neuropsychiatric disorders.

### Physiological versus psychogenic symptoms

In a recent editorial on a paper dealing with medically unexplained movement disorders, Stone and Edwards (2011) make the following comment:

Proving that a symptom affecting a heterogeneous group of people is “psychogenic” using epidemiological data may be as unachievable as trying to prove that it is not. The word psychogenic itself suggests an exclusion of biological factors that is at odds with a biopsychosocial model now prevalent in understanding most mental health disorders and physical symptom syndromes. The use of this term (i.e. psychogenic) may play a role in restricting research scope, and also may adversely affect clinical interest, care, and understanding of these patients. Studies such as this are helpful in challenging stereotypes, and may push us away from a narrow dogmatic approach and toward a broader view of the etiologies and mechanisms of these common and disabling disorders.

In this context, we discuss a number of questions related to CRPS and neuroinflammation. Specifically, does neuroinflammation underlie many of the sensory, autonomic, and movement disorders of CRPS? If so, why have these dysfunctions so often been interpreted as being psychogenic? What diagnostic and mechanistic criteria are being used to make these conclusions? Are the diagnostic criteria for psychogenic illness compatible with emerging views of neuroinflammation and neuroautoimmunity? In the following sections, we discuss how autoimmunity and neuroinflammation may combine to produce some of the most controversial symptoms in neurology and psychiatry: the co-morbidities of CRPS.

### Neuroautoimmunity and the initiation of CRPS

**Fig. 1:**
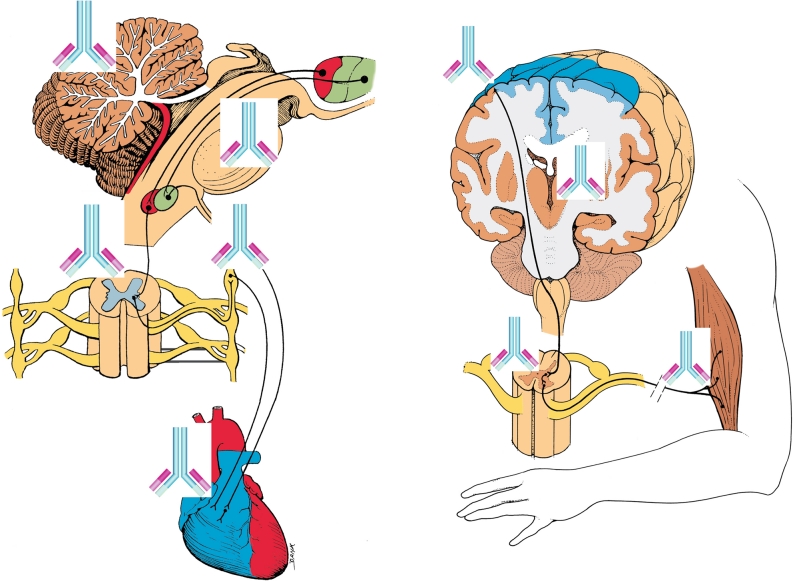
Potential autoantibody binding sites in CRPS patients for β2AR (beta(2)-adrenergic receptor) and M2R (muscarinic 2 acetylcholine receptor) autoautoantibodies. (images modified from The Inner Man™, Medical Illustrations Company LLC). **a**. β2AR and M2R sites in the central nervous system and heart: β2AR in cerebellum—(Reznikoff et al. 1986); β2AR in reticular formation—(Culmsee 2009); β2AR in sympathetic autonomic nervous system—(Katzung 2001), M2R in parasympathetic autonomic system and heart—(Katzung 2001) β2AR also in astrocytes and microglia (Mantyh et al. 1995; Wang et al. 2010) **b**. M2R distribution in pyramidal motor pathway to skeletal muscles M2R in motor cortex and thalamus—(Levey et al. 1991) M2R in peripheral nerves—(Katzung 2001)

Recent discoveries have helped to elucidate possible mechanisms for the initiation and progression of CRPS. In general, neuroautoimmune responses are determined by how infiltrating leukocytes react to autoantibodies, which bind to autoantigens located on the surfaces of neuronal and glial cell targets (Fig. 1). For a substantial fraction of adult CRPS cases (Kohr et al. 2011), initiation of CRPS may lie in a breakdown of immunologic self-tolerance, and the development of autoantibodies to the β2AR and M2R neurotransmitter receptors. Once autoantibodies have been generated to these neuroantigens in a given individual, an ongoing progression of stereotyped autoimmune-mediated neuroinflammatory responses might become initiated.

**Fig. 2:**
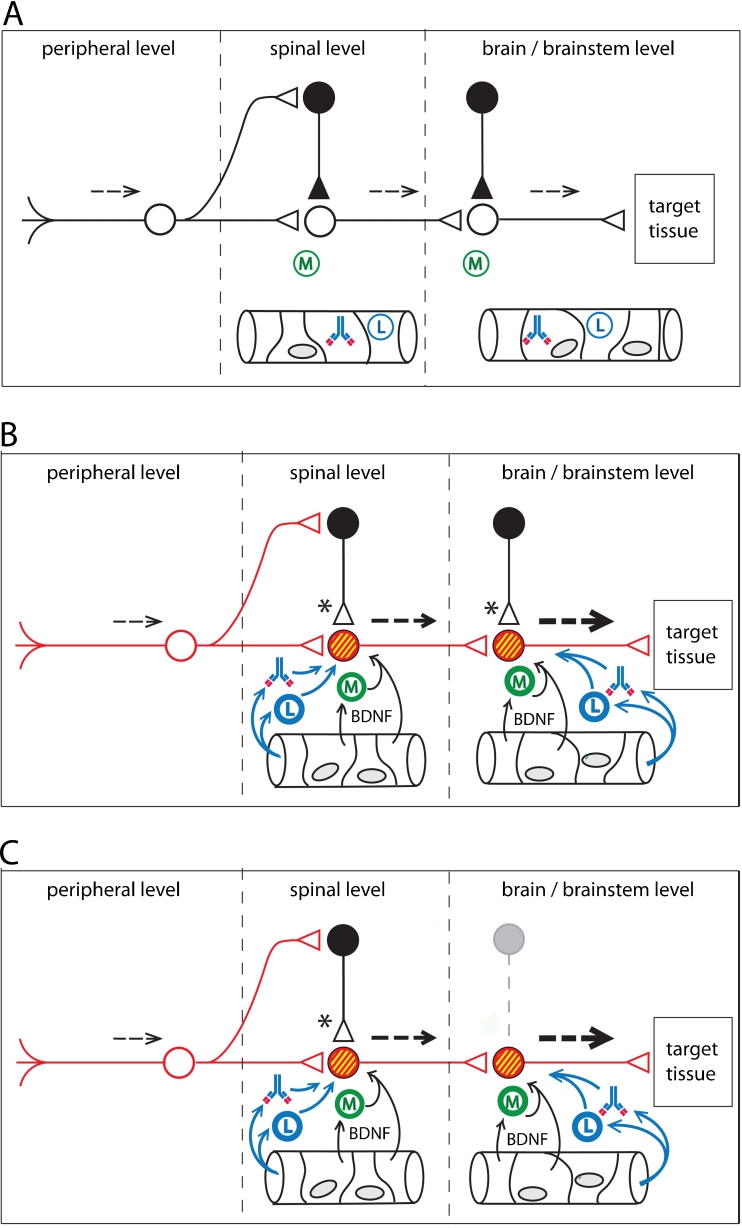
A conceptual model for functional pathophysiologies arising from neuroinflammatory tracks. Neuroinflammation leads to a loss of sensory gating, as well as excessive gain, in sensorimotor pathways. **a**. Normal sensory processing (dotted lines). GABAergic interneurons provide inhibitory tone at both first order and second order synapses. Non-activated microglia (M) lie in the CNS parenchyma. Leukocytes (L) and autoantibodies are present in the CNS vasculature. **b**. Peripheral injury initiates anterograde central sensitization. Microglial activation takes place in response to cytokines released at the terminals of injured primary afferents (first order synapse). Breakdown of the blood–brain barrier (or the blood-spinal cord barrier) allows activated leukocytes and autoantibodies to extravasate into the parenchyma of the CNS, leading to autoimmune mediated neuroinflammation. Release of Brain-Derived Neurotrophic Factor (BDNF) from plasma, endothelial cells, and activated microglia alter chloride homeostasis in post-synaptic neurons. Accumulation of intracellular chloride (yellow) results in synaptic conversion, changing GABAergic and glycinergic synapses from inhibitory tone to excitatory tone (Huberfeld et al. 2007; Price et al. 2009; Cooper and Przebinda 2011). Sensory gating is lost at the first order synapse. Graded sensory transmission is lost within the neural circuit. Upregulation (red) of excitatory transducers (e.g. glutamate receptors) results an increase in excitatory tone. Functional changes in the circuit result in a collapse of feedforward inhibition and filtering at the first-order synapse (pain gate in the nociceptive circuit) (Cooper and Przebinda 2011). Neuroimmune activation spreads to the second order synapse through the release of the cytokine CCL21 from the terminals of first order neurons (Saab and Hains 2009). Small sensory stimuli provoke large (thick dotted arrows) volleys in second order neurons. **c**. Central injury to the CNS can result in retrograde central sensitization via neuroimmune activation. Loss of inhibitory interneurons (grey outlines), either by autoimmune attack (e.g. Stiff-Person Syndrome) (Sandbrink et al. 2003; Rokocevic and Floeter 2012), or by metabolic crisis (e.g. hypoglycemia, hypoxia, or excitotoxicity), permanently reduces inhibitory tone in the sensorimotor circuit. These conditions, and/or the conditions illustrated in panel B, may contribute to post-traumatic dystonias, non-dermatomal pain distributions, autonomic, and/or somatovisceral disorders, in patients with autoimmune-mediated neuroinflammation, even after the initial neuroinflammatory event subsides

Achieving a cellular and molecular understanding of the clinical progression of CRPS, as well as the generation of its complex symptoms, requires modeling of the immunologic and integrative physiology involved. It is important to consider how distressed neurons and glial cells release factors that stimulate the extravasation of leukocytes and autoantibodies, from the bloodstream, into the parenchyma of the CNS (Watkins et al. 2007). Serious neuroinflammatory consequences would be expected to arise when β2AR and M2R autoantibodies exudate from blood vessels, together with complement proteins and leukocytes (Figs. 1 and 2).

Beggs et al. (2010) have recently found that the blood–brain barrier in the spinal cord of rats is transiently compromised in response to peripheral nerve injury. Extravasation of leukocytes into the parenchyma of the cord has been found to last for several days following a sciatic nerve injury (Milligan ED, personal communication). In a CRPS patient with a peripheral nerve injury, one might expect circulating autoantibodies to exudate into the parenchyma of the injured nerve. Autoimmune attack on peripheral nerves might trigger leukocyte extravasation, autoantibody exudation, and neuroimmune activation in the spinal cord as well. Neuroinflammation in the cord could produce a mixture of pain, autonomic dysfunctions, somatovisceral dysfunctions, and/or abnormal motor functions. CRPS is a neurological disorder where many of these dysfunctions can be expressed in a single patient (Schwartzman et al. 2009).

Vascular breakdown or leakage may be a critical step in allowing autoantibodies access to the neuroautoantigens of CRPS patients. Focal accumulations of neuroautoantibodies on target cells are likely to initiate neuroinflammatory responses. Whether the antibody-initiated neuroinflammation remains as discrete foci, or whether the neuroinflammation begins to propagate through the neuraxis, are key questions for understanding the chronicity and spread of CRPS symptoms in a given patient. CRPS shows distinctive patterns of spread throughout the body (van Hilten et al. 2001; van Rijn et al. 2011). Spread of CRPS symptoms to other body sites often occurs in a contiguous fashion. However, it is possible for CRPS symptoms to appear quickly in non-contiguous locations (van Hilten et al. 2001). Both types of CRPS spreading behavior could be linked to the establishment and spread of neuroinflammation within the neuraxis. At this point in time, how CRPS symptoms spread within the neuraxis is primarily known from an analysis at the symptom level. Cellular and molecular knowledge about the spread of neuroinflammation within the neuraxis in other disorders comes from the neuroimaging of human patients, as well as from animal model studies of neuropathic pain.

### Spreading neuroinflammation

Numerous studies have revealed that neuroinflammation does not always remain confined to specific foci in the CNS. Cascades of intercellular signaling allow neuroinflammation to migrate either anterograde or retrograde from primary sites of neuroinflammation.

Banati et al. (2001) utilized radiolabeled PK11195 as a biomarker to image spreading neuroinflammation in the CNS in response to peripheral nerve injury. They determined that peripheral nerve injury frequently leads to microglial activation at the first order synapse of injured neurons. In a minority of cases, neuroinflammation migrated trans-synaptically to the second order synapse, located in higher levels of the CNS, such as the thalamus. Banati (2002) also postulated that by altering neurotransmission and the coding of neuroinformation, neuroinflammation at the first and second order synapses could become drivers for higher-order functional changes within the nervous system.

In concise terms, Banati’s principles are:

- Neuroinflammation can spread to second order synapses via remote neuroimmune activation.
- Neuroinflammation may be the physiological driver for functional pathologies at third order synapses, as well as higher-order integrative processes (Banati 2003).

Hereafter, we refer to paths of spreading neuroinflammation in the CNS as *neuroinflammatory tracks* (Fig. 2). This term refers to discrete pathways of neuroinflammation that involve anatomical linkage between primary and secondary foci of neuroinflammation.

The ontogeny of posttraumatic neuroinflammatory tracks was first documented in rodents by Banati et al. (1997). Later, Banati et al. (2001) determined that a cohort of chronic pain patients, who had suffered an antecedent peripheral nerve injury, exhibited persistent microglial activation in their contralateral thalamus. Banati et al. (2001) proposed that neuroinflammation had migrated from first order synapses in the spinal cord in these patients, to second order nociceptive synapses, which are located within the contralateral thalamus. Banati (2003) have also obtained evidence for remote neuroimmune activation (also referred to as transsynaptic microglial activation) in a number of neurological disorders.

Saab and Hains (2009) have discussed cellular and molecular mechanisms for such remote neuroimmune activation. Release of the cytokine CCL21 from the terminal of first order nociceptive neurons is capable of initiating secondary sites of neuroinflammation where the nociceptive neurons terminate, such as the VPL nucleus of the thalamus. Saab and Hains (2009) have postulated the neuroinflammation in the thalamus drives dysrhythmias in thalamocortical loops, which can be detected using EEG (Saab, 2012), thus producing a central generator for neuropathic pain.

Neuroautoantibodies in CRPS patients could modify, exaggerate, and sustain this type of remote neuroimmune activation (Fig. 1). In this regard, remote neuroimmune activation via neuronal projections may account for the spread of microglial and astroglial activation within the spinal cord of CRPS patients (Del Valle et al. 2009; van Rijn et al. 2011). It has also been hypothesized that spinal neuroinflammation could spread to supraspinal sites in CRPS patients via remote neuroimmune activation to initiate movement disorders (Cooper and Przebinda 2011; Cooper 2011b).

### Neuroinflammation and central sensitization

The term “central sensitization” is often used to describe changes in the central nervous system associated with the establishment and progression of the neuroinflammatory diseases. In an experimental model of peripheral inflammatory pain (Roberts et al. 2009), neuroinflammation spreads from the first order synapse, located in the spinal cord, to a second-order synapse in the rostroventromedial medulla (RVM)**.** Sensitization of first order and second order trigeminothalamic neurons, for instance, may contribute to the ontogeny of migraine (Borsook et al. 2006; Noseda et al.^2011^). Non-dermatomal patterns of pain can be generated by neuroinflammation within the thalamus (Burstein et al. 2010).

In CRPS patients, features of central sensitization appear to involve a complex set of neuroinflammatory responses involving NMDA receptors, glial cell activation in the spinal cord (Kiefer et al. 2008; Del Valle et al. 2009), as well as the release of pro-inflammatory cytokines from neurons, glia (Munts et al. 2008), and leukocytes (Alexander et al. 2012).

Extravasation of leukocytes and exudation of autoantibodies into the parenchyma of the nervous system are key features of central sensitization during a neuroautoimmune attack of the CNS. The autoimmune attack is focused on sites where autoantibodies bind. Autoimmune attack can potentially lead to the loss of GABAergic inhibitory interneurons (e.g. Stiff-person Syndrome) (Rokocevic and Floeter 2012). In addition, BDNF released from endothelial cells (Bayas et al. 2002) or activated microglia in a neuroinflammatory site (Trang et al. 2012) can produce a downregulation of the potassium-chloride co-transporter, KCC2 (Blaesse et al. 2009; Trang et al. 2012), which plays a critical role is maintaining transmembrane chloride gradients in CNS neurons.

Reduction of the transmembrane chloride gradient in the post-synaptic neuron leads to conversion of inhibitory tone to excitatory tone (asterisks in Fig. 2). KCC2 downregulation appears to help BDNF promote neuronal survival (Huberfeld et al. 2007). By converting GABAergic and glycinergic synapses to excitatory function, BDNF promotes increased Ca^2+^ influx into the affected neurons. Although these dynamics may help neuronal survival (Huberfeld et al. 2007), the resulting synaptic conversion can potentially provoke major disruptions in the logic of neural circuits, leading to major dysfunctions in sensory, motor, and autonomic processes (Cooper and Przebinda 2011) (Fig. 2).

Major nerve injuries in rodents and humans are known to result in leukocyte infiltration into the PNS and CNS (Stoll and Bendszus 2010). Exudation of autoantibodies into the parenchyma of basal ganglia is hypothesized to initiate neuroinflammation and movement disorders (Ahlskog et al. 2001; Citak et al. 2004). Peripheral nerves injury can result in a transient breakdown of the blood-spinal cord barrier and the blood–brain barrier (Beggs et al. 2010). The transient breakdowns in these barriers can be blocked by local anesthesia, indicating that the transient increase in capillary permeability in the CNS is dependent on peripheral nerve activity.

Do minor peripheral nerve injuries in CRPS patients trigger the extravasation of leukocytes and the exudation of autoantibodies from blood vessels, leading to the initiation of persistent sites of neuroinflammation in the PNS, autonomic nervous system, and CNS? Later in this paper, we discuss whether modern neuroimaging methods will be able to answer this question.

### CRPS movement disorders

CRPS researchers have utilized symptomatology to create validated diagnostic criteria for CRPS (Harden et al. 2010). Using a set of four major symptom categories (amplified pain, autonomic dysfunction, motor dysfunction, sudomotor/trophic changes), a set of diagnostic criteria has been identified, which is both sensitive and specific to identify CRPS (Harden 2010). The movement disorders associated with CRPS include fixed dystonia, myoclonus, and tremors. Approximately 80% of CRPS patients have paresis in their affected body limbs (Birklein et al. 2000).

The nosological effort to define the movement disorders of CRPS as being either physiologic (organic) or psychogenic (non-organic) has been long and controversial (Reedijk et al. 2008; Munts and Koehler 2010). Recently, a group of movement disorder specialists, who have studied fixed dystonia in CRPS patients, have stated:

“Traditional medical dualism polarizes opinion as to whether fixed dystonia is best characterized as a psychogenic or an organic disorder. …On the basis of our cases and previous data with regard to amputation in complex regional pain syndrome type I (CRPSI), we speculate that fixed dystonia is itself part of the spectrum of body integrity identity disorders.”

Toward the end of their paper, Edwards et al. (2011) state:

“The theory outlined above generates some testable hypotheses regarding the pathophysiology of fixed dystonia that attempt to move beyond the dualistic battle between classifying these patients as organic or psychogenic toward a more integrated view of brain dysfunction in this enigmatic disorder.”

This statement represents a major conceptual shift in the differential diagnosis of fixed dystonia. The statement suggests that the fixed dystonias should be reanalyzed in terms of observable neuropathologies. Indeed, distortion of the body schema has been detected in CRPS patients using fMRI (Maihöfner et al. 2003, 2004, 2007). Deficient drive from the parietal cortex to the supplementary cortex and motor cortex is also detected (Maihöfner et al. 2007), leading to “neglect-like” motor symptoms (Galer et al. 1995; Kolb et al. 2012).

Psychogenic was originally coined to indicate non-organic phenomena “originating in the mind” (Lewis 1972). Using the original semantic of the term “psychogenic”, it is difficult to interpret distortions of somatosensory maps in CRPS patients as evidence for psychogenic disease. Evidence for body schema distortions, for instance, has been detected in patients with low back pain (Bray and Moseley 2011). Widespread allodynia can be produced in rodent models by inducing extensive neuroinflammation within the thalamus (Burstein et al. 2010). Thus, non-dermatomal pain distributions, or distortions of the body schema in the somatosensory cortex, cannot be used as positive diagnostic evidence for psychogenic illness. Body schema distortion or thalamic neuroinflammation provide physiological alternatives to the differential diagnosis.

The need of obtaining an accurate differential diagnosis for a disorder is different from the goal (and/or timescale) of obtaining a mechanistic explanation for a disorder. As a consequence, the clinical goals for obtaining a differential diagnosis of a disease state, versus obtaining a mechanistic explanation for the disease state, should not be conflated. In the absence of positive diagnostic evidence for a presumed etiology, the term “idiopathic” should be employed for the symptom, rather than “medically unexplained symptom.” This later term biases differential diagnosis towards the unwarranted conclusion of psychogenic illness. From a nosological perspective, it is unclear whether movement disorders that are linked to neuroinflammation should be classified as organic movement disorders, functional movements disorders, or both.

### Re-conceptualizing CRPS symptoms

Can the symptoms of CRPS be re-classified in terms of mechanism/processes, rather than presumed etiology? Without a complete knowledge of how symptoms develop with time, perhaps it is most effective to elucidate whether specific physiological states are present in a given patient. In terms of evaluating neuroautoimmune mechanisms for CRPS symptoms, it is useful to consider five interconnected processes: (a) autoimmune seroconversion, (b) amplification of autoimmune cells and autoantibodies, (c) autoimmune attack, (d) neuroimmune activation, and (e) functional and structural disruption of neural networks.

CRPS symptoms might be categorized by using one or more of the following mechanistic processes and concepts, which could result in the type of lesions indicated:

- Infiltration of autoantibodies into nervous tissues (functional lesion)
- Infiltration of leukocytes into nervous tissues (structural/functional lesion)
- Focal sites of cytokine imbalance (functional/dynamic lesion)
- Remote neuroimmune activation of glia (structural/functional lesion)
- Breakdown of blood brain barrier (structural lesion)
- Loss of inhibitory tone (functional lesion)
- Excessive loop gain in neural circuits (dynamic lesion)
- Thalamic neuroinflammation (structural/functional lesion)
- Loss of sensory gating (functional lesion)
- Synaptic conversion (functional lesion)
- Thalamocortical dysrhythmias (dynamic lesion)
- Distortion of a somatotopic map (structural/functional lesion)
- Altered connectivity within the brain (dynamic/functional lesion)

By utilizing the concepts of neuroinflammatory lesions, dynamic lesions, and functional lesions, the differential diagnosis focus shifts away from establishing a psychogenic versus physiologic etiology for a given symptom, toward a more mechanistic analysis of the multiple functional and structural pathologies that are common in CRPS patients.

### Linking neuroautoimmunity with sensorimotor dysfunction

Neuroinflammation provides a wide range of mechanisms to alter sensorimotor function. A recent publication describes a form of chorea in three individuals with nonketotic hyperglycemia (Wang et al. 2009). The authors hypothesize that circulating autoantibodies enter the parenchyma of the basal ganglia as a consequence of hyperglycemia shock to the microvasculature, resulting in a breakdown of the local brain-brain barrier. Microvascular lesions in the basal ganglia were detected with MRI and gadolinium contrast. In one patient the lesion expanded from the right globus pallidus to include the right putamen (Wang et al. 2009):

“These results, together with distinctive distribution of the lesions affecting the putamen, globus pallidus and the head of the caudate without involving the anterior limb of internal capsule, suggest that the autoimmune-mediated inflammatory process against basal ganglia neurons may take part in the pathogenesis of some patients with NKH (nonketotic hypoglycemia) induced HC**–**HB (hemichorea-hemiballism). Conceivably, the basal ganglia might be susceptible to autoimmune attack by anti-GAD65 or other autoantibodies via opening of the blood–brain barrier by hyperglycemia-related hyperosmolality.”

The movement disorders in these patients might also be caused by immune complexes activating microglia in the basal ganglia to produce pro-inflammatory cytokines, which in turn induce neuronal dysfunction.

The authors hypothesize that neuroinflammation in these patients results from vascular lesions. Localized breakdown of the blood–brain barrier allows autoantibodies to infiltrate into the parenchyma of the basal ganglia. In this context, *the neurovascular lesion is both an organic lesion, as well as a functional lesion.* Structural features of the neurovascular lesion are often visible by MRI. Functional changes produced by the neurovascular lesion are evident in the development of the movement disorder. In this case, the neurophysiological mechanisms by which autoimmune-mediated neuroinflammation in the basal ganglia creates the functional pathology (*i.e.* hemichorea) remain unknown. Microvascular lesions in the brain and spinal cord may be a common way for individuals with neuroautoimmune disorders to have transient neurological and neuropsychiatric events, including movement disorders (Mayer et al. 2010).

The above case illustrates the inherent difficulty of using “medically unexplained symptoms” as a positive diagnostic term. In the previous example, positive radiological evidence provides a plausible etiology of the movement disorder, as well as a positive explanation for the positive response to pharmacological intervention. However, what would the clinical diagnosis be if MRI were not available, and an actual neurovascular lesion were not visible with X-ray or CT? In the absence of evidence for such structural changes, a practitioner applying the DSM-IV diagnostic criteria could easily misdiagnose the patient as having a motor conversion disorder. Thus, the clinical diagnosis, as well as the subsequent treatment scheme, hinges on the inherent instrumental sensitivity (sometimes marginal) of the neuroimaging modality used to detect neurovascular disruption.

### Autoimmunity and movement disorders

Neurologists and psychiatrists have commented that certain individuals appear to be pre-disposed to certain disorders. Some of these individuals may have autoimmune disorders (Appenzeller et al. 2011; Vincent et al. 2011; Irani and Vincent 2011). Unusual tardive movement disorders might occur in individuals with autoimmunity. The following study is illustrative.

A cohort of female patients with antiphospholipid antibodies who took oral contraceptive drugs developed chorea (Cervera et al. 1997). The chorea ceased once the medication was discontinued. The authors of this paper concluded that the unique combination of antiphospholipid (aPL) antibodies and oral contraceptives in these patients produced a movement disorder. The authors hypothesized that chorea arose because of changes in blood flow and metabolism in the basal ganglia, produced by the entrance of autoantibodies into the parenchyma of the basal ganglia. This hypothesis of a neuroautoimmune-mediated metabolic disturbance fits well with the functional lesion concept, originally posited by Charcot (1885) to explain the etiological origins of “hysteria.”

Instead of autoimmune-mediated neuroinflammation, the term “medically unexplained symptoms” has often been used to describe sensory, autonomic, and motor symptoms of CRPS patients, even though there is an extensive literature describing neurophysiological pathologies and mechanisms in these patients. There are several inherent difficulties with the psychogenic hypothesis. However, absence of evidence is not evidence of absence. In addition, characteristics such as the speed of onset and remission have also been used to assign symptoms to neurological vs. psychogenic causes, with the assumption that neurological disease processes have a longer timecourse than psychogenic ones. In this regard, rapid onset and rapid remission have often been used as criteria for diagnosing psychogenic movement disorders, even though autoimmune disorders are well know for their cyclic character of remission and relapse.

Another salient difficulty in psychogenic hypotheses is that they often conflate a cluster of atypical symptoms with a psychological pathology. This reasoning excludes atypical presentations of physiological processes, including neuroinflammation and neuroautoimmunity, from the differential diagnosis. A patient may have an atypical genetic background, leading to atypical symptoms. Alternatively, the person may have a latent immune response to an infectious agent (Söderberg–Nauclér 2012). Or the person may have been placed on a medication that is counter-indicative for a nervous system with focal sites of neuroinflammation, resulting in anomalous motor or sensory phenomena. In addition, prior injuries, surgeries, infectious antigen presentations, or autoantibody production may have ‘primed’ the neuroimmune system for an exaggerated neuroimmune response (Hains et al. 2010; Frank et al. 2012). Any or all of the above conditions may exist in certain CRPS patients. These alternative etiologies should be considered before assigning a putative psychogenic etiology to any particular symptom. Self-limiting chorea, for instance, has been associated with Parvovirus B19 infection in children (Fong and de Sousa 2006; Grillo and da Silva 2009). In these cases, immune-mediated encephalopathies are thought to underlie the chorea, even though the MRI is unremarkable in these patients (Grillo and da Silva 2009).

In the diagnosis of CRPS, symptoms such as fixed dystonia are often considered to be a “conversion disorder” (Verdugo and Ochoa, 1995, 2000; Hawley and Weiner 2011). Rather than being a conversion disorder of a psychological origin, these fixed dystonic postures may be the functional consequences of a “seroconversion” event, with autoantibodies serving as the initiators of neurological and neuropsychiatric symptoms. Thus, the autoimmune response itself may have been induced by a combination of physical trauma(s) and hormonal changes associated with psychological stressors, perhaps the onset of symptoms themselves, which further enhance the autoimmune response and symptom severity.

Health professionals have often described physical trauma as antecedent events for CRPS, since the condition was first described (Mitchell 1872). However, the role for psychological stressors as an initiator of CRPS, rather than a consequence of the disorder, has not yet been substantiated (de Rooij et al. 2010).

Individuals with CPRS have often been viewed as having a pre-existing susceptibility for exaggerated pain and movement disorders. If true, where does the predisposition originate? One possibility is that this predisposition is related to the presence of autoantibodies to β2AR and M2R. Neuroautoantibodies, for instance, have been found to be associated with Sydenham’s chorea (Pavone et al. 2006; Brilot et al. 2011), Sjögren's Syndrome (Vincent et al. 2011), as well certain cases of Tourette’s Syndrome (Müller et al. 2004; Morer et al. 2008; Dehning et al. 2009). CRPS may have a similar etiology.

Autoantibodies have been shown to be of creating multiple pathologies within the body (e.g. Sjögren's Syndrome). Memory B-cells, and the autoantibodies that they produce, could be the encoded memory of an earlier traumatic event. Autoantibodies can be generated from immune responses to neoplasms (Vincent et al. 2011). The key concept here is that the development of autoimmunity to specific neuroautoantigens may be the initiating event for many cases of CRPS. Psychological stressors, physical trauma, infectious agents, and/or genetic susceptibility could all play a role in the breakdown of self-tolerance, and the onset of an autoimmune response. This set of etiological linkages fits well with documented clinical experience with CRPS (Mitchell 1872; Birklein et al. 2000). Psychological stressors and immunologic priming have been linked to the enhanced activation of microglia to nervous system injury (Frank et al. 2007; Hains et al. 2010).

### Imaging neuroinflammation in movement disorder patients

Autoimmune-mediated movement disorders are thought to occur in a number of autoimmune disorders, such as Sjögren’s Syndrome (van den Berg et al. 1999; Venegas et al. 2005; Papageorgiou et al. 2007; Min and Youn 2009), Sydenham's chorea (Vincent et al. 2011), and certain cases of Tourette’s Syndrome (Morer et al. 2008; Dehning et al. 2009). Methodologies currently exist that may provide clinically useful means to image neuroinflammation in these and other disorders. Using such methods, neuroinflammation might be expected to be found using PET or MR spectroscopy in the basal ganglia of aPL patients, and in these and other neural fields in Sjögren's Syndrome, Sydenham's chorea, and Tourette’s Syndrome, among others. It also would be expected that the degree of symptoms, such as tics or chorea, would correlate with the degree of neuroinflammation.

What happens if the predictive biomarkers are “functional changes” in the nervous system? Neuroimaging and neurovisualization methods are now providing such functional biomarkers to identify neurophysiologic disorders. Changes in blood flow in the brain are commonly used to distinguish alterations of connectivity within the brain. In certain cases, regional changes in brain metabolism can be correlated with transient movement disorders (e.g. onset of paroxysmal or itcal dystonias) (Joo et al. 2004; Yoon et al. 2007).

The issue of sensitivity is central to the clinical tractability of using neuroimaging methods to discern the effects of neuroinflammation in movement disorders. In the case of immune-mediated movement disorders, Citak et al. (2004) have commented:

… in our patients with tics, there were no abnormalities on CT or MRI. On the other hand, similar to the patients with Sydenham’s chorea, our patients with post-streptococcal tic disorder also showed striatal hypoperfusion in their SPECT images. This might be due to the fact that structural imaging studies might not be as sensitive as functional imaging studies for detecting the abnormalities in striatum in Sydenham’s chorea and tic disorders.

The difficulty in detecting certain neuroinflammatory lesions in the brain using structural MRI is also illustrated in the following case study (Maeda et al 2010). A diabetic man exhibited a transient hemiballism (lasting 4 days) in his right arm, which was linked to a hypoglycemic crisis. The movement disorder remitted in response to glucose stabilization with insulin, as well as hemodialysis. Death occurred two months after the transient hemiballism, from severe autonomic neuropathy and cardiac arrest. After autopsy, immunohistological analysis revealed activated microglia in the man’s left subthalamic nucleus (STN). Within the same STN, no evidence of neurodegeneration or activated astroglia was found. It is not clear whether activated microglia played a causative role in the movement disorder. Microglial activation was present in the STN, while the hemiballism was in remission. This illustrates that activated microglia alone cannot explain the transient hemiballism in this patient. However, the presence of activated microglia in the STN contralateral to the affected limb does suggest that a physiological event did occur in the STN, perhaps leading to a persistent activation of microglial in the affected STN. Notably, the patient’s brain MRI remained unremarkable during these transitions (Maeda et al. 2010).

This above case study strongly supports Charcot’s prediction (1885) that dynamic and functional lesions in the CNS could serve as drivers for movement disorders. In the above case of hemiballism, the patient apparently suffered from a functional lesion that had a neuroinflammatory component, without appreciable neuronal death within the STN. However, the neuroinflammatory, functional lesion could not be visualized using standard MR imaging techniques (Maeda et al. 2010).

Cell death within the CNS is, of course, a possible outcome of hyperglycemic or hypoglycemic shock. Neuronal cell death may explain why some patients do not experience resolution of their chorea after their metabolism is normalized (Ahlskog et al. 2001, as irreparable damage may have already occurred (Shan et al. 1998). Loss of inhibitory interneurons from autoimmune attack (Rokocevic and Floeter 2012) can contribute to excessive excitability in the excitatory neuron population of deep brain nuclei. This potential outcome to injury and/or neuroinflammation is illustrated is Fig. 2c. Autoimmune-mediated movement disorders are thought to occur in a number of autoimmune disorders, such as Sjögren's Syndrome (van den Berg et al. 1999; Venegas et al. 2005; Min and Youn 2009), Sydenham's chorea (Vincent et al. 2011), and certain cases of Tourette’s Syndrome (Morer et al. 2008; Dehning et al. 2009).

### Neuroinflammation and movement disorders

Movement disorders occur in a number of disorders that include neuroautoimmune components, such as Sjögren's Syndrome (van den Berg et al. 1999; Venegas et al. 2005; Min and Youn 2009; Alonso-Navarro et al. 2009), Sydenham’s chorea (Brilot et al. 2011; Pavone et al. 2006), and certain cases of Tourette’s Syndrome (Morer et al. 2008). Autoimmune-mediate neuroinflammation of the basal ganglia is suspected in many of these cases.

Besides autoimmune-mediated movement disorders, peripheral trauma has long been known to be an antecedent of certain movement disorders (Mitchell 1872; Sankhla et al. 1998; van Rooijen et al. 2011). Mitchell (1872) was among the first to recognize a linkage between neuroinflammation and the genesis of movement disorders in CRPS. Although evidence linking neuroinflammation and movement disorders is rapidly accumulating (van de Warrenburg et al. 2007; Simonyan et al. 2010; van Rooijen et al. 2011), it is clear that a complementary set of neuroimaging methods will be needed to visualize the cellular players and metabolism of neuroinflammatory lesions, to determine their putative roles in motor dysfunction. Neuroinflammatory lesions are clearly heterogeneous in their cellular dynamics, their metabolism, as well as their chronicity.

An idiopathic case of hyperekplexia (startle syndrome) is instructive in this regard (van de Warrenburg et al. 2007). A 55-year-old woman initially presented with a stiff neck, as well as retching when she placed a toothbrush into her mouth. These symptoms resolved over a period of two months. The patient subsequently developed bilateral jaw aches when chewing food. This was followed by severe involuntary mouth closure on attempts to eat. Two months later, she noticed that her arms and shoulders would jerk in response to unexpected sounds or on touching her face. One month later, her left arm became stiff and tended to assume abnormal postures. Her gait became more effortful and she tended to veer to the left.

The patient developed upper and lower extremities jerks, as well as trismus. In addition, the patient developed ptosis on the right side. Decreased pinprick sensation around the nose, upper and lower lip, and forehead; tight and overactive masseter muscles with incomplete mouth opening. There was also rigidity and dystonic posturing of the left arm with mild distal weakness; jerks that were elicited by taps on nose, jaw, forehead, crown and neck and that consisted of bilateral shoulder abduction and forearm flexion with additional posturing of the face. Following symptomatic treatment with medications, most brainstem abnormalities gradually ceased. However, the hyperekplexia persisted for 10 years. The timecourse and characteristics of the patient’s symptoms were interpreted as a possible case of self-limiting brainstem neuroinflammation (van de Warrenburg et al. 2007). The authors also suspected that the neuroinflammation was autoimmune-mediated.

The movement disorder symptoms of the above patient have important similarities to certain cases reported by Sims et al. (2012) (see Movies S3 (fixed dystonia that remits) and S4 (paroxysmal dystonic attacks) in this paper). These cases, as well as the specific pattern of progression of symptoms in the above case of idiopathic symptomatic hyperekplexia, may involve migration of neuroinflammation from sites of peripheral nerve injury into the brainstem. The specific symptoms suggest that the trigeminal nerve may be involved. Several papers have documented the onset of cranial dystonias following dental procedures or other trauma to tissues in the oral cavity (Sankhla et al. 1998; Schrag et al. 1999). Migration of neuroinflammation from an injured branch of the trigeminal nerve into first-order and second-order synapses located in the brainstem might serve as drivers for the genesis of such movement disorders (Sims et al. 2012).

Brainstem neuroinflammation was previously considered to explain a case of hyperekplexia, apparently starting from an insect bite on the lower right leg (Kellett et al. 1998). In this patient, movement disorders involving the right leg occurred before generalized body spasms. Migration of neuroinflammation from the spinal level to the reticular formation might provide a mechanism to explain the chronicity and progression of these symptoms. Kellett et al. 1998 postulated a pathology involving glycinergic synapses in the brainstem. A collapse of transmembrane chloride gradients in post-synaptic neurons (Cooper and Przebinda 2011), resulting from neuroinflammation in the brainstem, might account for a loss of glycinergic tone and GABAergic inhibitory tone (Fig. 2c), possibility producing symptomatic hyperekplexia.

### Ascending and descending neuroinflammation

As discussed earlier, the formation of neuroinflammatory tracks, as well as a concomitant the loss of inhibitory synapses and/or interneurons, may allow certain neural pathways to become sensitized centrally. Post-traumatic dystonias and CRPS dystonias might evolve in this manner (Cooper 2011a). Neuroinflammation spreading to second-order synapses in supraspinal centers provides a potent mechanism to destabilize feedback circuits, such as those involved in proprioception, nociception, and autonomic functions. Prolonged sensitivity may result from the loss of inhibitory tone in the affected neural pathway (Rossi et al. 2012).

Remote neuroimmune activation could establish secondary foci of neuroinflammation in the motor cortex (Cooper 2011a), reticular formation, red nucleus, thalamus, cerebellum, rostral ventrolateral medulla (RVM), periaqueductal grey (PAG). Once inhibitory tone is diminished in two or more locations, decompensation is likely to occur, leading to excessive gain in both feedforward and feedback neural circuits (Prescott et al. 2006; Cooper and Przebinda 2011).

In addition to excessive gain in nociceptive, autonomic, and motor circuits, timing errors could also occur in these pathways. Loss of inhibitory tone at the cellular level could easily lead to abnormal time-to-fire behaviors in proprioceptive neurons. This could lead to errors in limb perception, as well as the regulation of postural tone. Body schema distortions might also be generated by neuronal coding problems. We suggest here that neuroinflammatory tracks may be especially potent in producing information processing errors. In feedback systems, two interacting sites within the neuraxis, each with neuroinflammation, may be unable to compensate for corrupted information produced in the other site. Neuroinflammation driven abnormalities in neuronal firing and timing could potentially result in a host of symptoms, which are now being termed functional sensory and motor disorders, instead of psychogenic disorders (Edwards et al. 2012). Such functional sensorimotor integration dysfunctions are prevalent, and sometimes extreme, in CRPS patients (Schwenkreis et al. 2005; McCabe and Blake 2007; Lewis et al. 2007; Turton et al. 2007; McCabe et al. 2009; Huge et al. 2011; Cohen et al. 2012). How specific symptoms are viewed and labeled can dramatically influence the course of medical inquiry, patient-practitioner (biosocial) relationships, as well as the patient acceptance of treatment plans (Stone and Edwards 2011; Edwards and Bhatia 2012).

**Fig. 3:**
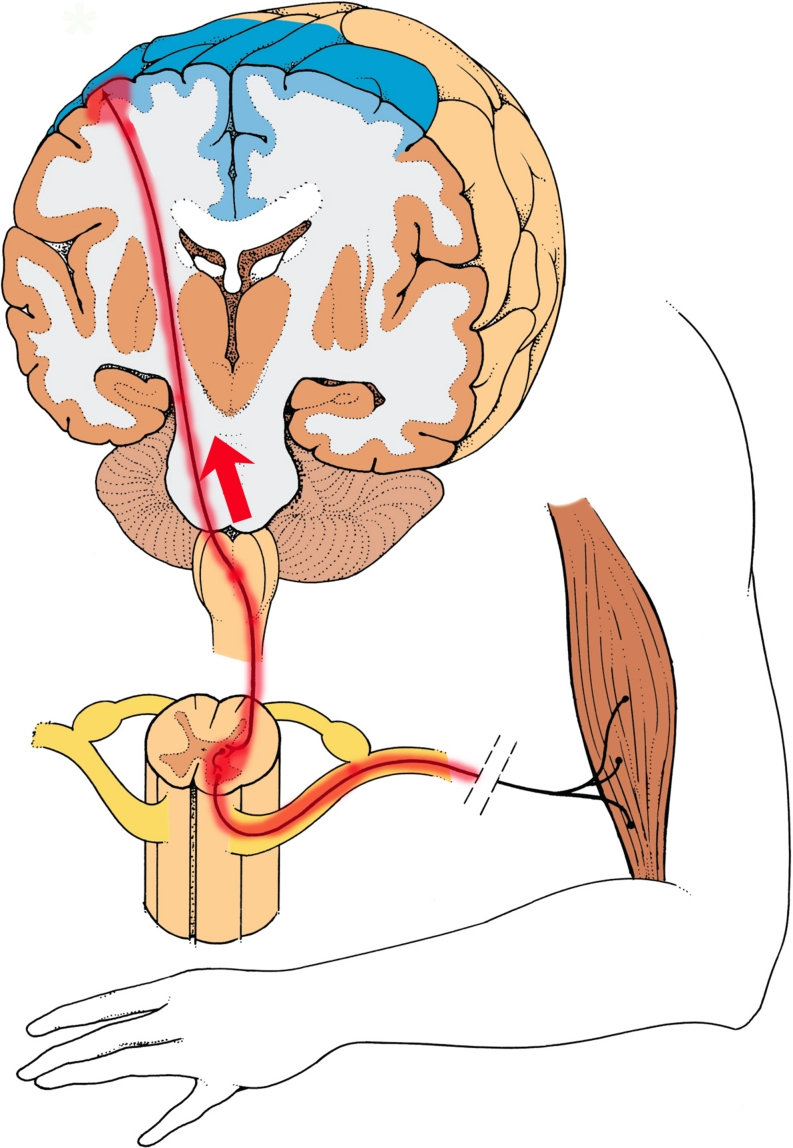
A neuroinflammatory model for the generation of fixed dystonia. Injury to a peripheral nerve results in inflammation of motor neurons. Neuroinflammation is established in the spinal cord, ipsilateral and segmental to the peripheral nerve injury (Sigel et al. 2007; Lee et al. 2009). Retrograde migration of neuroinflammation (red arrow) occurs via corticospinal neurons, leading to a secondary neuroinflammation site in the motor cortex (Cooper 2011a). Loss of chloride-dependent inhibitory tone (similar to panel 2B) and/or an increase in excitatory tone in the cortical and spinal neuroinflammatory foci lead to tonic firing of segmental motor neurons. A neuroinflammation-mediated loss of intracortical inhibition in the motor cortex may also be required for the genesis of fixed dystonia. (images modified from The Inner Man™, Medical Illustrations Company LLC)

Changes in muscular tone, ranging from paresis to fixed dystonia, may occur in an affected extremity of CRPS patients (Birklein et al. 2000; van Hilten 2010). Such changes have long been viewed as stigmata for “hysteria”, psychosomatic, or psychogenic illness. The dystonias of CRPS and other fixed posttraumatic dystonias need to be reanalyzed from the standpoint of neuroinflammation states. Multi-site information coding failures could easily occur in neural circuits affected by multi-focal neuroinflammation. Substantial evidence has been obtained that spinal reflex circuits are disinhibited at the spinal level in CRPS dystonia (Schouten et al. 2003; van de Beek et al. 2002). Loss of chloride-dependent inhibitory tone at the spinal level, resulting from neuroinflammation, could account for some of this disinhibition. We suggest here that an additional loss of inhibitory tone at supraspinal sites, such as the motor cortex (Cooper 2011a; Fig. 3), reticular formation, and/or red nucleus, might be needed to maximally decompensate spinal motor reflex circuits, and produce fixed dystonia (Cooper MS (2011b).

Whether neuroinflammation spreads from spinal sites to supraspinal sites in CRPS patients is currently unknown. However, if neuroinflammation does spread from the spinal level to supraspinal sites in CRPS patients, disinhibition of excitatory neurons could occur at both levels, leading to tonic activation of motor neurons (e.g. Fig. 3). This could potentially produce tremor, myoclonus, or fixed dystonia, depending upon the dynamics and interactions of different motor control centers. Diminished intracortical inhibition has been detected in individuals with posttraumatic fixed dystonias (Espay et al. 2006).

Entrapped nerves are a known etiology of certain cases of cervical and focal dystonias (Ross et al. 1995; Charness et al. 1996; Alafaci et al. 2000; Sun et al. 2009). Neuroinflammatory tracks, with both peripheral and central neuroinflammatory contributions, may play central roles in mediating the ontogeny of these peripherally induced dystonias. To test these hypotheses, it may be soon possible to visualize the distribution and chronicity of both central and peripheral neuroinflammation in movement disorder patients, using a complementary set of emerging neuroimaging methods.

### Cytokines and changes within the connectome

Standard neurological examinations are carefully constructed to detect structural defects within the nervous system (Clark et al. 2009). When aberrant reflexive or higher-order neurological behaviors are detected, but cannot be immediately linked to defined structural defects in the nervous system, the aberrant symptoms are often classified as functional pathologies. This distinction, however, is confounded when these pathologies are linked to neuroinflammatory lesions, because neuroinflammation can simultaneously generate both structural and functional changes within the nervous system. Drivers for such changes can include altered cytokine production in peripheral tissues, infiltrating leukocytes, and non-neuronal components of the CNS (glia and endothelial cells) can each become drivers for such changes (Bayas et al. 2002; Ledeboer et al. 2005; Cao and DeLeo 2008; Milligan and Watkins 2009).

It is well established that pro-inflammatory cytokine expression is linked to chronic pain (Milligan and Watkins 2009). Pro-inflammatory cytokines are also associated with depression, fatigue, sleep disorders, and executive dysfunction (Barbosa et al. 2012; Blume et al. 2011; Capuron and Miller 2011; Mondal et al. 2008). In animal models of neuropathic pain, IL-1β expression becomes elevated in the brainstem, prefrontal cortex, and hippocampus (Apkarian et al. 2006; Besedovsky and del Rey 2011; del Rey et al. 2011). Elevation of IL-1β RNA expression in the hippocampus suggests a direct modulation of a site that is also involved in depression and anxiety (del Rey et al. 2011). In addition, it has been reported that an increase in IL-1β expression occurs in the contralateral hippocampus after unilateral sciatic nerve injury (Apkarian et al. 2006).

This later result, along with other earlier reports (Banati et al. 2001; Banati 2002; Roberts et al. 2009), supports the concept that neuroinflammation can become established in supraspinal centers after peripheral nerve injury. Spreading neuroinflammation provides a non-psychogenic etiology to plausibly explain the progression and chronicity of certain disease states, as well as the migration of symptoms to different portions of the body. This mechanistic concept of spreading neuroinflammation within the CNS needs to be incorporated into differential diagnosis of neurological and neuropsychiatric disorders, as well as into the standard use of the Diagnostic and Statistical Manual for Mental Disorders (DSM). This would substantially advance the recognition and diagnosis of neuroinflammatory-mediated functional disorders within the biomedical community.

Cytokine production is a central part of the overlapping and interlocking mechanisms that produce neuroinflammatory disorders, including CRPS. Neuroendocrine alterations and disturbances in the metabolism of monoamines have been correlated with cytokine-induced mood and cognitive symptoms (Capuron et al. 2002; Dantzer et al. 2008; Doorduin et al. 2009; Engler et al. 2012). In addition, neuroimaging studies have revealed alterations in specific brain regions during cytokine treatment (Kullmann et al. 2012; Schneider et al. 2012). These changes could contribute to the development of fatigue, depression, and cognitive alterations in patients with neuroinflammation (Raedlera 2011; Schedlowski 2012). In CRPS patients, fatigue, paresis, depression, anxiety, and cognitive impairments are well documented (Mitchell 1872; Birklein et al. 2000; Apkarian et al. 2004).

Numerous structural and functional changes take place in neural networks as a result of altered cytokine expression. To describe effects of neuroimmune activation in neurological and neuropsychiatric disorders, it is important to consider how neuroinflammatory lesions and cytokines affect the *connectome,* the map of neural connections within the nervous system (Sepulcre et al. 2012). It is also useful to view this connectome as being part of a larger neuromatrix (Iannetti and Mouraux 2010), which is modulated by cellular communication between with a variety of cellular players, including glia, endothelial cells, and leukocytes (Milligan and Watkins 2009; Hughes et al. 2012). Cytokines help mediate these cellular interactions, and thus modify the activities of multi-partiate synapses (i.e. synapses between multiple cell types) within the neuromatrix (De Leo et al. 2006).

At the cellular level, neuroinflammation can produce structural lesions in the following ways: demyelination of axonal fibers (Staff et al. 2010); stripping of synapses from cell bodies by microglia (Perry and O’Connor 2010; Yamada et al. 2011); autoimmune attack on specific interneurons (Rokocevic and Floeter 2012); sprouting of afferent axonal terminals; dieback of sensory nerve endings (Oaklander and Fields 2009); altered dendritic aborizations (Jakubs et al. 2008). These microscopic structural lesions lead to functional changes of local neuronal circuits. Although these structural changes within the connectome may remain largely undetectable using clinical neuroimaging methods, the functional/behavioral changes that result from these structural changes can be profound. For instance, small fiber neuropathies in the cornea are correlated with the occurrence of painful blepharopasm (Borsook and Rosenthal 2011). In an experimental study, large-fiber neuropathies in peripheral nerves have been correlated with the onset of fixed dystonic postures (Siegel et al. 2007). Clinically, peripheral nerve injury is a known antecedent for many forms of dystonia (Jankovic 2001; 2009), including the fixed dystonias seen in CRPS II patients (50% of the cohort) (Birklein et al. 2000).

In the CNS, activated microglia can strip neuronal cell bodies of synapses and dendrites, as well as promote demyelination (Perry and O’Connor 2010; Yamada et al. 2011). These microstructural lesions might help to explain the persistent changes in pain processing in children with a history of CRPS, months after their allodynia has remitted (Lebel et al. 2008). In post-surgical inflammatory neuropathies, reversible demyelination can occur (Staff et al. 2010). Weakness and pain disappear in the affected extremities as remyelination proceeds. The cellular behaviors that underlie demyelination and remyelination should be considered when dealing with the transient expression of neurological and neuropsychiatric symptoms, as well as their long-term remission.

From a number of neuroautoimmune disorders, it is known that the pathological ramifications of neuroautoantibodies entering the parenchyma of the nervous system can be profound. Autoantibodies to β2AR in CRPS patients, for instance, could bind to β2AR receptors located on microglia (Mantyh et al. 1995; Wang et al. 2010), leading to autoimmune attack on these neuroimmune cells, as well as specific types of neurons and astrocytes, which also express β2AR. Aberrant cytokine production from activated microglia is likely to occur if these neuroimmune cells are subjected to an autoimmune attack mediated by autoantibodies and infiltrating leukocytes.

CRPS patient have elevated levels of the pro-inflammatory cytokines IL-1β and IL-6 in their cerebrospinal fluid, as well as reduced levels of the anti-inflammatory cytokines IL-4 and IL-10 (Alexander et al. 2005; Alexander et al. 2005). In peripheral tissues, pro-inflammatory cytokines are found in the affected limbs of CRPS patients (Schinkel et al. 2006), and are enhanced upon transcutaneous electrical stimulation, indicating that neurogenic inflammation is present (Birklein and Schmelz 2008). In general, it is known that antidromic firing of C-fibers (driven by both axonal reflex and dorsal root reflex) can result is CGRP (calcitonin gene related peptide) and SP (substance P) release from the peripheral endings of C-fibers (Willis 1999; Hagains et al. 2010). CGRP and SP sensitize mast cells and the endings of C-fibers. At the peripheral level, local cytokine production can also drive the progression of neuroinflammatory pathologies in many target organs (Li et al. 2009b; Lia et al. 2009). MicroRNA levels are also altered in the blood of CRPS patients (Orlova et al. 2011). These miRNA biomarkers potentially offer a new way for monitoring inflammation in CRPS patients, as well as stratifying patients for therapeutic interventions.

A number of CRPS co-morbidities, including visceral pain, osteopenia, edema, and rashes and ulcerations of the skin, may involve prolonged neurogenic inflammation, as well as its pathological consequences (Oaklander and Fields 2009). Through its corruption of circuit logic within the connectome (Cervero and Laird 1996; Prescott et al. 2006; Cooper and Przebinda 2011), neuroinflammation in spinal, supraspinal, and autonomic centers, might also generate unusual modulations of these diverse pathologies (e.g. episodic or diurnal expression of symptoms). However idiosyncratic the presentation of symptoms may be in individual CRPS patients, it is increasingly difficult to assert a purely psychogenic etiology for the co-morbidities of CRPS. The structural and functional consequences of neuroinflammatory lesions provide a logical framework for conceptual modeling and clinical investigation, during the differential diagnosis of unusual neurological or neuropsychiatric symptoms.

### Promising neuroimaging approaches

A MRI-based method that may eventually provide a means to diagnose neuroinflammation is magnetic resonance spectroscopy (MRS). 1H-MRS measures small changes in proton resonance that result from shielding by orbiting electrons. Depending on the chemical structure of the substance being measured, its resonance signature may be detected, and its amplitude (i.e. concentration) can be quantified.

Using this method, a variety of neurochemicals can be identified, including various neurotransmitters and their byproducts (glutamate, glutamine and GABA) membrane components (myo-inositol) constituents of metabolic pathways (choline and creatine) and others with no well understood purpose, but that are sensitive to neuronal function and damage (n- acetyl aspartate, abbreviated as NAA). MRS has been used in a variety of studies to examine chemical changes related to disease processes that may involve neuroinflammation (Holt et al. 2012).

Aside from 1H-MRS, hyperpolarized 13C-MRS provides a means to label carbon atoms in specific metabolically active molecules, which can be injected and are taken up into physiological processes in the body (Rothman et al. 2002). While not radioactive, 13C can produce an NMR signal that is small, but detectable. The imaging advantage of 13C is being able to focus on specific metabolic pathways, such as GABA and glutamate, and the ability to quantify their concentrations and changes in concentration as other variables are manipulated. These methods have been used pre-clinically to detect tumors and tumor metabolism. Hyperpolarized 13C-MRS has yet to be utilized to image neuroinflammation in human patients.

As physical properties of the CNS change with neuroinflammation, it might be possible to visualize them using non-invasive imaging methods that depend only on intrinsic properties of the CNS. Using methods such as MRI, the difficulty is often not in the degree of sensitivity, but in specificity. Especially in the case of MRI, the measures that can be obtained are affected by a large variety of factors, and so may differ from the population mean due to a variety of reasons, many of which may reflect properties not involved in the disease process. The specific causes of changes found in MRI data from the CNS can be difficult to infer. Changes in the number or size and type of any CNS cell could contribute to these structural changes, including the size and numbers of either different classes of neurons or glia, as well as their metabolic state (Shan and Pan 2007).

While intriguing, such effects are also often inconclusive as to their mechanisms. Additionally, due to a high degree of individual variability, many imaging protocols require that data be obtained before and after the eliciting event or some intrinsic change in the CNS, which provides a much more sensitive basis to detect effects compared with what is essentially a between-subjects comparison that is found in most physician offices. These two features: (a) lack of specificity and (b) lack of sensitivity for a single imaging sample in an individual patient, remain major barriers to using imaging to identify neuroinflammatory lesions in clinical practice. What is needed is to identify measures that are both sensitive and specific, and can be obtained from a single imaging session.

Recently, magnetic nanoparticles have been used in human patients to detect the infiltration of leukocytes into injured nervous tissues (Stoll and Bendszus 2010; Thorek et al. 2011; Deddens et al. 2012). New contrast agents are being developed for PET/SPECT (Chopra et al. 2012), MRI (Stoll et al. 2012), and MRS imaging (Li et al. 2009a; Witney and Brindle 2010) as well. MRS and diffusion tensor imaging (DTI) can also be used to examine changes in nervous tissues affected by factors such as chronic viral infection (Holt et al. 2012). These rapidly advancing technological developments may also allow neuroinflammation to be imaged in difficult sites, such as the brainstem and spinal cord. Such neuroimaging studies would be hugely beneficial for understanding the establishment and spread of CRPS symptoms.

## Conclusion

In clinical practice, when an organic explanation cannot be found for functional disorders, psychogenic etiologies are often asserted. However, as the understanding of functional disorders improves, it appears that neuroimmune and neuroinflammatory disorders are much more common than previously thought. Neuroautoimmunity combined with neuroinflammation together provide a viable etiology for the relapsing-remitting chronicity, atypical presentation and intensity of neurological and psychiatric symptoms.

When faced with medically unexplained symptoms, health practitioners and patients should actively seek physiological hypotheses, as an alternative to diagnoses of somatoform or conversion disorders. Neuroinflammatory lesions, along with their functional consequences, should always be considered in the differential diagnosis of medically unexplained symptoms, even when commonly used clinical neuroimaging protocols fail to reveal positive evidence of neuroinflammation.

When viewing the symptoms of an individual patient, health practitioners should anticipate that a given patient might have a complex, multifocal pattern of neuroinflammation and associated functional symptoms. The presentation of symptoms may be idiosyncratic to that patient, depending on the topographic pattern and severity of neuroinflammation and other variables, often yet undiscovered. However, key mechanistic elements of spreading neuroinflammation could be common to a wide spectrum of patients and disorders. By integrating neuroinflammation and neuroautoimmune concepts into the differential diagnosis of neurological and neuropsychiatric symptoms, a number of functional sensory and motor disorders may become less medically unexplainable than was previously thought. As diagnostic methods continue to improve, it may someday be possible for neuroautoimmuity and neuroinflammation to be commonly assessed as a part of a differential diagnosis of chronic pain syndromes such as CRPS.
